# Prognostic impact of adjuvant chemotherapy treatment intensity for ovarian cancer

**DOI:** 10.1371/journal.pone.0206913

**Published:** 2018-11-12

**Authors:** Kristen D. Starbuck, J. Brian Szender, William D. Duncan, Kayla Morrell, John Lewis Etter, Emese Zsiros, Kunle Odunsi, Kirsten Moysich, Kevin H. Eng

**Affiliations:** 1 Department of Gynecologic Oncology, Roswell Park Comprehensive Cancer Center, Buffalo NY, United States of America; 2 Department of Biostatistics and Bioinformatics, Roswell Park Comprehensive Cancer Center, Buffalo NY, United States of America; 3 Department of Cancer Prevention and Control, Roswell Park Comprehensive Cancer Center, Buffalo NY, United States of America; National Cancer Center, JAPAN

## Abstract

**Objective:**

We aimed to investigate the prognostic impact of duration of first-line chemotherapy administration in patients with epithelial ovarian cancer (EOC).

**Methods:**

Chemotherapy records were abstracted from the electronic medical record. Patients with on-time completion (105 days) were compared to patients finishing early (<105 days), delays of 1–4 weeks, or >4 weeks. For 222 women with stage IIIC/IV, stage-stratified estimates of progression-free survival (PFS) and overall survival (OS) were compared. A delay sub-study was performed with outliers removed. Each week of delay was correlated with the change in PFS and OS to identify time points associated with change in outcome.

**Results:**

Most women had on-time completion of chemotherapy (23.6%) or a treatment delay of ≤4 weeks (21.8%); 21.6% of women experienced a delay longer than 4 weeks. R0 resection at initial debulking (OR = 1.99, 95%CI: 1.18–3.36, p = 0.010) and RECIST complete response (OR = 4.88, 95%CI: 2.47–10.63, p<0.001) were strongly associated with on-time completion. Patients with on-time completion and < 1 month delay had similar median survivals of 43.1 months (lower 95% CI bound 33.7 months) and 44.5 months (lower bound 37.0, p = 0.93). Women with >1 month delay had decreased median survival of 18.1 months (14.7–24.9 months), while women with short intervals survived 35.0 months (95%CI: 21.8–49.8 months). Short-term delays lead to progressively decreasing OS. This was significantly different from the on-schedule survival estimate after 6 weeks of delay.

**Conclusions:**

On-time completion of chemotherapy correlates with increased survival and higher complete response rates. Increasing delays in chemotherapy completion were associated with decreased survival.

## Introduction

Epithelial ovarian cancer (EOC) is the most lethal gynecologic malignancy. In 2017, it was estimated that there will be 22,440 new cases of ovarian cancer and an estimated 14,080 people will die of this disease[1]. Unfortunately, increasing knowledge of the biology of ovarian cancer has not translated into significant gains in overall survival. Presently, clinicopathologic factors, including age, performance status, FIGO stage, histologic grade, residual disease after initial cytoreductive surgery, and response to chemotherapy; are the strongest prognostic features known to affect survival.

Important elements related to chemotherapy include platinum sensitivity, duration of previous response, and potentially, delay in the initiation or duration of chemotherapy administration. Exclusive of neoadjuvant regimens, delays can arise after primary debulking surgery but prior to chemotherapy or during adjuvant chemotherapy. Studies of the impact of treatment delay on survival generally focus on delays to the initiation of chemotherapy.

The effect of chemotherapy delays and dose reductions on progression free and overall survival in the treatment of epithelial ovarian cancer has been documented in several retrospective studies. One of the first large analyses by Wright et al.[2] looked at delays in the time to initiation of chemotherapy after surgery using SEER-Medicare data on patients with advanced EOC undergoing radical cytoreductive surgery. They found that receipt of chemotherapy 12 weeks or more after surgery resulted in a significant increased risk of death (HR = 1.32, 95% CI:1.07–1.64). This effect was similar in magnitude to, but independent of, multiple complications from surgery.

A large dataset analysis using the National Cancer Database[3] evaluated delay in initial chemotherapy administration after surgery. Patients included from 1998–2011, and delay was defined as delay in time to first administration of chemotherapy more than 28 days from surgery. In addition, the study found that the 25–29 day interval had the weakest association with death; it was not statistically different than the referent group (21–28 days from surgery). In the covariate analysis, initiation of chemotherapy after 36 days was significantly associated with a 14% increased risk of death.

Tewari et. al. published a post-hoc analysis of GOG 218 investigating the impact of time from initial debulking surgery to initiation of chemotherapy (carboplatin, paclitaxel, with or without bevacizumab) on overall survival[4]. The strengths of this study include large patient numbers (n = 1718), randomized study design, and detailed follow-up. The median time to surgery between groups was equal between treatment groups at 31 days. The analysis revealed that risk of death increased sharply if chemotherapy was initiated more than 25 days after surgery; effect was most pronounced for patients with R0 cytoreduction[4].

There is much less information on delaying the completion of adjuvant therapy. The classic platinum plus taxane adjuvant course of chemotherapy includes six cycles with a single infusion every 21 days, or a schedule of 105 days from first to last infusion. Delays to this schedule arise from common hematologic and infectious side effects that necessitate delays and dose reductions in chemotherapy regimens, in addition to non-medical indications for delay such as holidays, weather-induced clinic closure, and personal schedule preferences.

Patterns of chemotherapy administration were recently investigated in a cohort of 157 elderly (older than 65) patients with ovarian cancer[5]. The authors found that any chemotherapy delay decreased survival (HR = 1.75, 95%:1.09–2.82) on the order of 4.0 years (no dose delays) to 2.5 years (any dose delay). Although not stage stratified (84% were high stage), this result remained significant after controlling for age, stage, number of cycles received, and residual disease status. Patients experiencing two or more dose delays had a further reduction in survival with a median of 1.7 years (p<0.03). Conversely, patients who had dose reductions only, but no delays, did not experience decreased survival (p = 0.41)[5].

One study investigated the effect of dose reductions and/or delays and found no association with PFS or OS for any group[6]; however, the number of patients in each group was small and it is difficult conclude whether the p-values were due to a true lack of association or insufficient power.

In this study, our objective was to identify the amount of time delay in chemotherapy administration during upfront therapy that exerts an impact on patient outcomes, as assessed by progression-free and overall survival. We leveraged our electronic medical record (EMR) implementation and an ongoing study of patients at our cancer center to obtain high-quality and detailed chemotherapy administration data. We applied an exploratory framework to study the effect of delays in chemotherapy onset and in chemotherapy completion in both semi-parametric and non-parametric methods.

## Materials and methods

### Ovarian cancer cohort study

**Fig 1:**
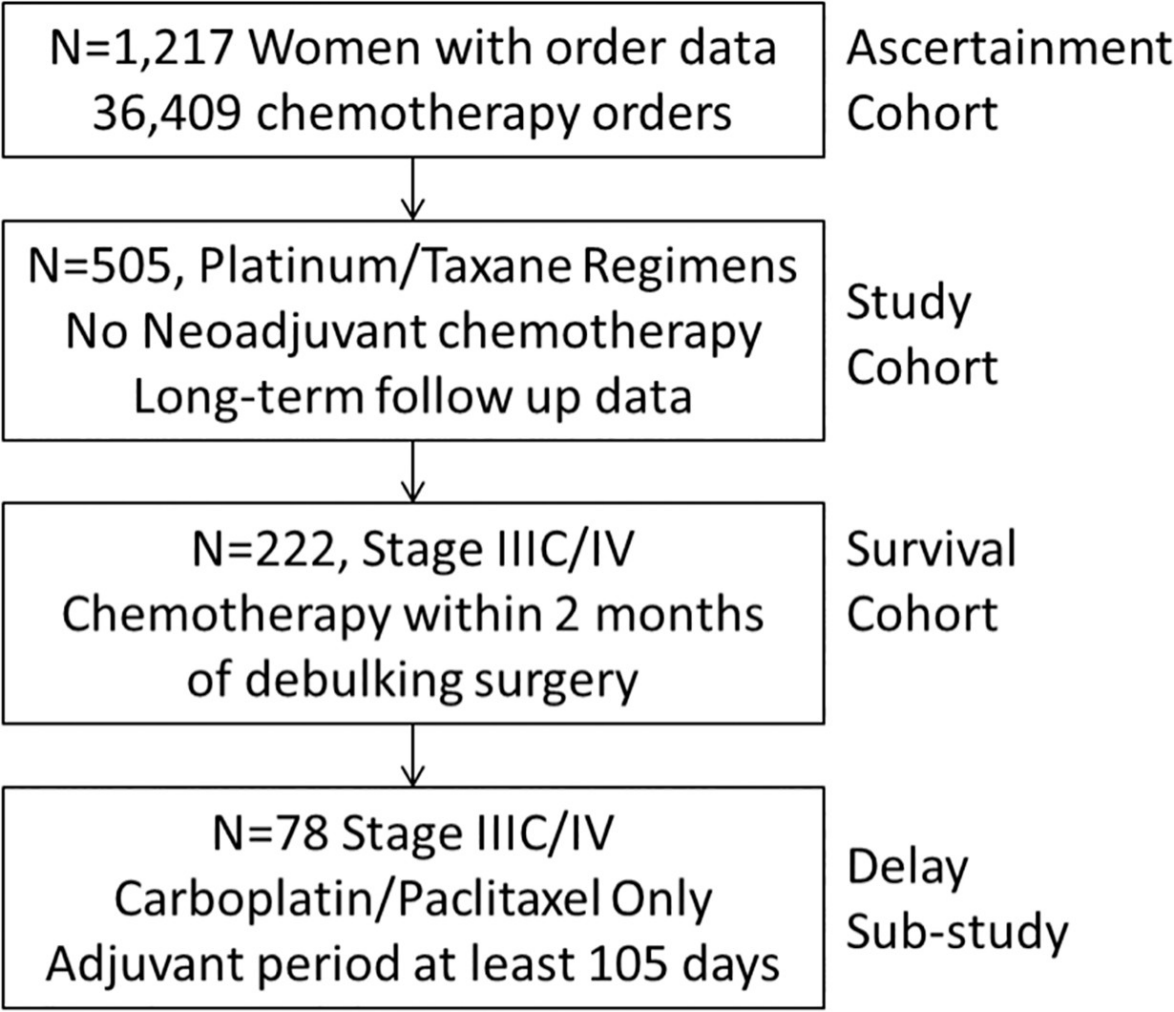
Study flowchart.

Following approval from the Roswell Park Comprehensive Cancer Center (RPCCC) IRB, we isolated 36,409 completed infusion orders for 1,217 women with primary ovarian cancer infused between May 2006 and December 2016. The patients under study consented to research follow up under IRB study I215512, and the institution has implemented universal research consent. For this project we additionally requested and were granted exempt research status with our IRB (BDR 054814) for the chemotherapy timing data during which identifiable data were accessed and abstracted. These women are a subset of the RPCI Ovarian Cancer Study previously described [7, 8]; briefly, these women underwent primary debulking surgery and completed their adjuvant regimens at our institution. We identified N = 505 women who received platinum/taxane adjuvant regimens, did not receive neo-adjuvant chemotherapy, and had long-term follow up data (through December 2017); we focused on these patients for the remainder of the study (Fig 1).

### EMR abstraction and data management

Chemotherapy treatment data was extracted from the hospital’s EMR. Using structured query language (SQL), we queried the relevant tables in the EMR for the chemotherapy agents used and treatment dates. Adjuvant interval timing was inferred from date of completed infusion orders.

### Survival analysis

Survival times were defined as the number of days from the end of adjuvant chemotherapy to death (censored by end of clinical contact). Progression-free survival is defined as the time from end of adjuvant chemotherapy to relapse or progression as defined previously [7]. Because the censoring rate for low stage cases was 76.7%, we did not continue survival analyses for these cases. The stage IIIC and IV censoring rate was acceptable at 37.4%. The continuous analysis uses a smoothing spline estimate with criterion-based smoothing[9].

### Statistical methods

All analyses were performed in R3.3.1 using the survival package. Tests were two-sided and specific tests are described within the results. Survival analyses use the Kaplan-Meier survival function to estimate medians and the log-rank test to compare the survival curves. Summaries of the regression models employ the restricted mean survival curve[10] with the corresponding pointwise 95% confidence interval. Note that the mean and median estimates are often different for survival time data; in this study, the mean is often longer than the commonly reported median. We choose to work with the mean in our sub-analyses because it had straightforward variance estimates. Adjusted odds-ratios are based on estimates from multivariate logistic regression.

## Results

### Initial chemotherapy agents

The majority of adjuvant platinum and taxane regimens were completed without changing agents (411/505, 81.4%). For these constant regimens, carboplatin/paclitaxel was the most common pair (381/411, 92.7%) with cisplatin/paclitaxel the second (19/411, 4.6%) and carboplatin/docetaxel (11/411, 2.7%) the third. For patients switching drug regimens, 5 patients switched drugs two or more times; most changes were to replace paclitaxel with docetaxel (65/94, 69.1% of all changes), the balance replaced cisplatin with carboplatin (24/94, 21.3% of all changes).

Other chemotherapy agents added to the adjuvant platinum regimen were bevacizumab (52/505, 10.2%), gemcitabine (40/505, 7.9), doxorubicin (22/505, 4.4%), and topotecan (13/505, 2.6%). Agents used in fewer than 1% of cases included megestrol, amifostine, etopositde, 5-FU, raloxifene, nivolumab, ifosfamide, cyclophosphamide, mercaptopurine, cetuximab, bleomycin and anastrozole.

### Time-to-complete adjuvant chemotherapy

The typical platinum-based adjuvant regimen is scheduled for exactly six 21-day cycles or a total of 105 days from the first to the last infusion. An “on-schedule” completion was frequent (119/505, 23.6%) with an equal number of women who experienced a treatment delay up to 4 weeks (110/505, 21.8%) or longer than 4 weeks (109/505, 21.6%). The balance of women finished their adjuvant intervals early (167/505, 33.1%) (Table 1). Women with treatment regimens longer than 210 days (48/505, 9.5%) reflected the long term use of regimens containing bevacizumab (29/45, 60.4%), gemcitabine (19/45, 39.6%), doxorubicin (13/45, 28.9%), and/or topotecan (12/45, 26.7%). A fraction of these women are likely to be GOG-218 participants treated with maintenance bevacizumab for 16 additional cycles[11].

**Table 1:** Patient characteristics by chemotherapy times.

	Time to complete Adjuvant Chemotherapy			
	< 105 days	105 days	> 105 days	p-value
N	167	119	219	
Age, mean	60.4	58.7	61.5	0.25
Primary Site				0.007
Ovary	128 (76.7%)	87 (73.1%)	156 (71.2%)	
Primary Peritoneal	19 (11.4%)	15 (12.6%)	41 (18.7%)	
Fallopian Tube	11 (6.6%)	2 (1.7%)	9 (4.1%)	
Multiple	9 (5.4%)	15 (12.6%)	13 (5.9%)	
Stage				0.001
I/II/IIIA/B	82 (49.1%)	62 (52.1%)	75 (34.2%)	
IIIC or IV	85 (50.9%)	57 (47.9%)	144 (65.8%)	
Grade				0.50
Well/Moderate	34 (22.8%)	26 (23.9%)	37 (18.8%)	
Poor/Undifferentiated	115 (77.2%)	83 (76.1%)	160 (81.2%)	
Histology				0.02
Serous	95 (56.9%)	65 (54.6%)	155 (70.8%)	
Clear Cell	11 (6.6%)	7 (5.9%)	6 (2.7%)	
Endometrioid	9 (5.4%)	11 (9.2%)	6 (2.7%)	
Mixed	11 (6.6%)	14 (11.8%)	19 (8.7%)	
Mucinous	4 (2.4%)	7 (5.9%)	6 (2.7%)	
Other	20 (12.0%)	10 (8.4%)	17 (7.8%)	
Debulking Surgery				
Optimal	88 (52.7%)	86 (72.3%)	136 (62.1%)	0.003
Suboptimal	79 (47.3%)	33 (27.7%)	83 (37.9%)	
Minimal Residual Disease				
R0	33 (19.8%)	37 (31.1%)	36 (16.4%)	0.006
Not R0	134 (80.2%)	82 (68.9%)	183 (83.7%)	
Chemotherapy Response*				< 0.001
Complete	78 (46.7%)	87 (73.1%)	104 (47.5%)	
Not Complete	67 (40.1%)	22 (18.5%)	92 (42.0%)	
Unevaluable	22 (13.2%)	10 (8.4%)	23 (10.5%)	
Platinum Response				<0.001
Sensitive	58 (72.5%)	64 (81.0%)	76 (55.9%)	
Resistant/Refractory	22 (27.5%)	15 (19.0%)	60 (44.1%)	
Months Median from Surgery				
Progression Free Survival	21.5	37.4	19.5	0.001
Overall Survival	55.3	85.3	46.5	0.001
Months Median after Adjuvant				
Progression Free Survival	18.7	34.0	15.3	< 0.001
Overall Survival	53.0	81.8	40.2	< 0.001
Months Median Survival				
After Relapse	17.2	31.0	19.3	0.13

*: RECIST Evaluation complete response after end of adjuvant chemotherapy

### Effect of time to adjuvant chemotherapy start on prognosis

We evaluated whether time between surgery and the initiation of chemotherapy influenced prognosis. In all stage data, the median time to start was 1.38 months (mean 4.89, 3^rd^ quartile 2.0 months). This suggested that over 75% of patients start chemotherapy within 2 months of surgery. This long tail was associated with low risk cases (overall survival 68.4 months versus 47.1 months, stage stratified, Log-rank p = 0.089) reflecting treatment selection bias. We removed these patients from subsequent survival analyses.

### Effect of time-to-completion on treatment success

Initial debulking to no visible residual disease (R0) was strongly associated with on time chemotherapy completion (OR = 1.99, 95%CI: 1.18–3.36, Fisher’s exact test p = 0.010) with 34.9% of R0 cases completing therapy in exactly 105 days. Similarly, patients evaluated as RECIST complete response at the end of their chemotherapy treatment were highly likely to complete chemotherapy on time (OR = 4.88, 95%CI: 2.47–10.63, p<0.001, histology and R0-adjusted OR = 6.10, 2.6–17.0, p<0.001) with an 83.0% complete response rate versus 54.6% for patients who experienced delays. This effect persisted through early follow up as we noted on time patients were more platinum sensitive (OR = 2.59, 95% CI:1.41–5.00, p = 0.002; histology and R0-adjusted OR = 3.7, 95%CI: 1.75–8.35, p<0.001.

### Effect of time-to-completion on prognosis

**Fig 2:**
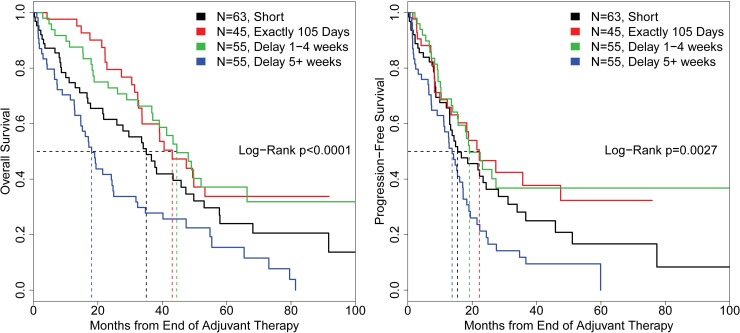
Stage-stratified Kaplan-Meier estimates of OS and PFS demonstrate long delays in chemotherapy (decreased chemotherapy intensity) and shortened chemotherapy regimens (incomplete regimens) are associated with poor outcomes.

Stage-stratified estimates of overall survival (OS) after completion of chemotherapy suggested that both long delays and short chemotherapy regimens were associated with poor prognosis (overall log-rank p<0.001) (Fig 2): women finishing chemotherapy on schedule survived 43.1 months (median, lower 95%CI bound 33.7 months) while women with short intervals survived 35.0 months (95%CI: 21.8–49.8 months), delays less than 1 month were not different from on time completion (44.5 months, lower bound 37.0 months, log-rank p = 0.93 vs. on time). Women with long delays survived a median of 18.1 months (14.7–24.9 months). The effect on progression-free survival (PFS) was similar (Fig 2): on time completion median PFS was 22.2 months (lower bound 13.5 months) with significantly shorter PFS for short and long delays (19.1 and 13.8 months respectively, log-rank p = 0.0027).

### Effect of time-to-completion and number of cycles delivered on prognosis

Considering the subset of Stage IIIC or IV patients with complete data, we cross-tabulated time-to-completion with the number of platinum infusions to quantify number of cycles delivered. Table 2 shows the predominant patterns: patients who completed treatment early received fewer than 6 cycles (N = 76 / 250, 30.4%); exactly 20% (N = 50/250) completed 6 cycles exactly on time; about 21.2% (N = 53/250) experienced a short delay in their 6 cycles; 8.4% had a long delay of 5 or more weeks (N = 21/250). The short adjuvant patients might be interpreted as patients who failed front-line therapy while on treatment. Of note, the patients receiving exactly 6 cycles showed an increasingly poor prognostic trend with additional delay.

**Table 2:** Interaction between number of cycles and the time to complete adjuvant chemotherapy.

	Time to Completion			
Number of Adjuvant Cycles	<105 days	105 days	1–4 week delay	5+ week delay
1–5 cycles	N = 76			
	HR = 1.73			
	p = 0.024			
	Median OS = 34.1			
6 cycles		N = 50	N = 53	N = 21
		HR = 1.00	HR = 1.35	HR = 2.15
		(Reference)	p = 0.270	p = 0.0225
		Median OS = 47.5	Median OS = 41.4	Median OS = 24.9
>6 cycles				N = 50
				HR = 2.42
				p = 0.0004
				Median OS = 21.5

Stage IIIC/IV only, Combinations with N<20 observations are suppressed

Age adjusted HR from Cox regression, Median OS from Kaplan-Meier estimate

### Estimating the effect of delay

**Fig 3:**
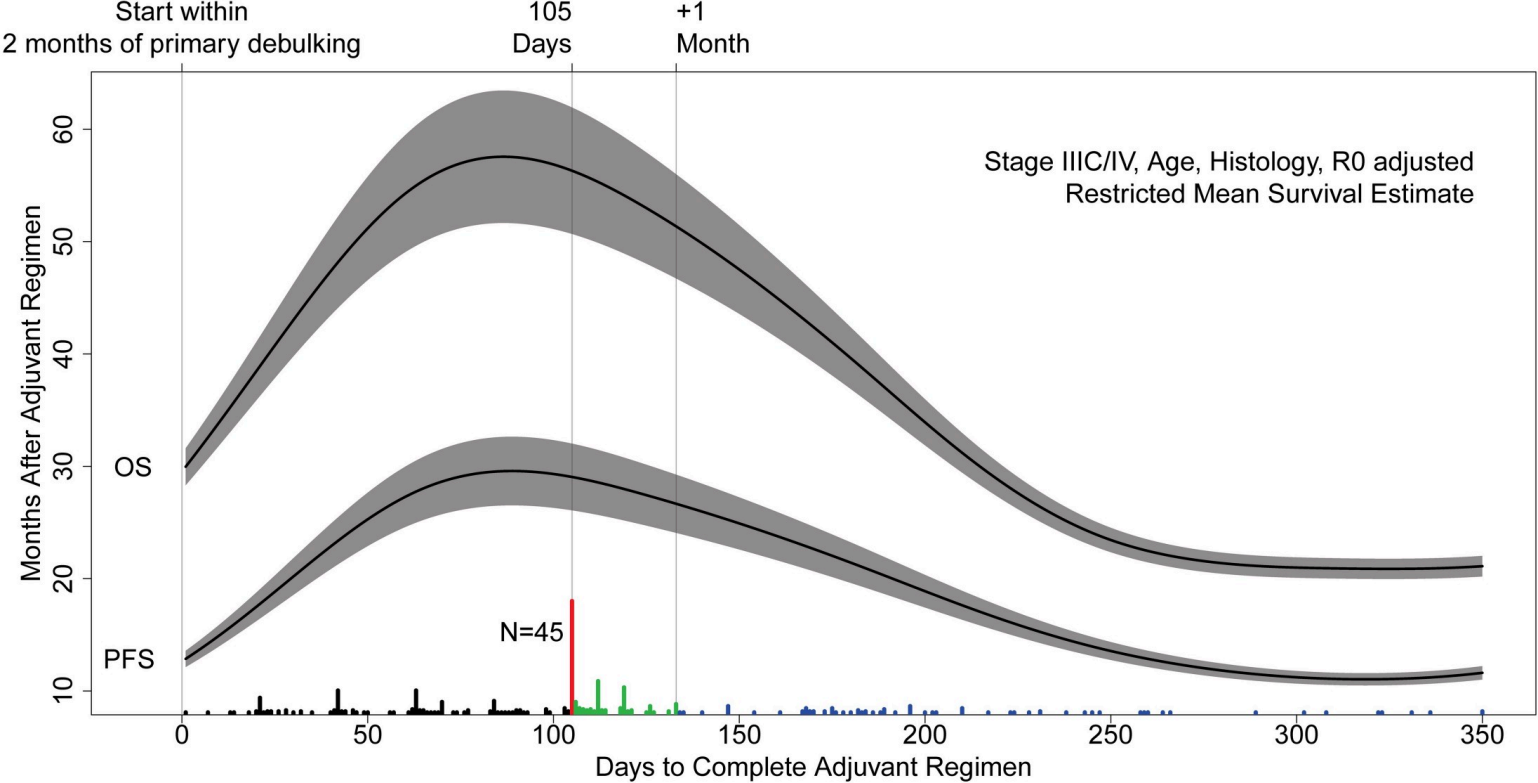
Continuous time-to-completion functional analysis of length of chemotherapy regimen and survival.

The Kaplan-Meier estimate does not permit a continuous interpretation of delay and may be simplistic as it implies that even a single day’s delay means at least a few months of lost survival. To better understand the timing of treatment delays, we analyzed women who experienced a delay using continuous time-to-completion. We fit a non-parametric Cox-proportional hazards model using a spline to estimate the functional relationship between the length of the adjuvant regimen and survival after its completion. This model was adjusted for stage, histology, and debulking status (as R0 status). Both the linear and non-linear parts of the functional estimate were associated with overall survival (score test p = 0.001) and progression-free survival (score test p = 0.03) implying a complex relationship best described visually (Fig 3). The survival loss associated with delay was clearly driven by extremely long delays: delays greater than 200 days to completion or about twice as long as planned. Again, the best prognosis was coincident with completing chemotherapy exactly on schedule. While the shape of the functional estimate suggested that prognosis may depend on a threshold in days of delay, it smoothed over the first month period. So, we investigated this with more granular estimates.

### Defining the delay threshold for prognosis

Based on the previous analyses, we determined that on time completion was associated with improved outcomes and that there was a clear loss of survival for intermediate and long delays. Using patients with the on-time completion as a reference, we stratified patients with delays into four equal and mutually exclusive groups for stable statistical estimates. Table 3 shows that the quarter of patients with a 1–8 day delay (1 week) have clinically significant reduced OS and PFS (6 and 3 months respectively) but the effect was not statistically significant. Progressive delays were associated with worsening OS, which became statistically significant after 105 days.

**Table 3:** Effect of treatment delay, categorized by quartiles.

	N (%)	Median OS (95% CI)	Median PFS (95% CI)
On time completion	N = 50	47.5 (39.1-NA)	16.6 (12.3–22.2)
1–8 day delay	N = 27 (21.8%)	41.4 (28.8-NA)	13.5 (10.3–23.2)
8–52 day delay	N = 32 (25.8%)	36.8 (18.5-NA)	12.0 (9.4–20.7)
52–105 day delay	N = 34 (27.4%)	32.4 (19.0–62.0)	13.3 (11.5–18.7)
> 105 day delay	N = 31 (25.0%)	18.1 (14.7–43.0)*	12.9 (8.1–17.2)*

Stage IIIC/IV with complete data

NA: Median estimate not achieved

CI: Confidence Interval

% based on patients who experienced a delay

*: Kaplan-Meier p<0.05 (Delay category vs. On Time)

**Fig 4:**
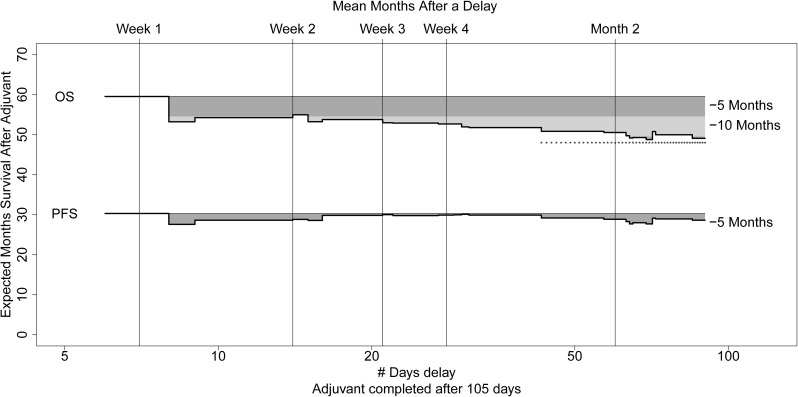
Delay sub-study: Effect of delays on survival by week in women receiving Carboplatin/Paclitaxel adjuvant chemotherapy with duration≥105 days.

Alternatively, we attempted to model the best threshold for prognosis. In addition, to address the potential for oversmoothing, we subset the data to N = 98 women who had neither extremely long nor short treatment intervals, completing therapy after 105 days. We considered survival time using women completing therapy on schedule as a referent category. We then considered the effect of adding in women with a given delay and estimated mean survival (RMS) plotting the changing estimate on a day-to-day basis to avoid parametric assumptions. As the delay grows (Fig 4), overall survival but not progression-free survival decays by 5 and 10 months. While these differences are statistically significant from the on schedule survival estimate after 6 weeks of delay, there is a clinically significant trend appearing with as little as 2 weeks of delay.

## Discussion

In the present study, we reviewed an observational cohort to understand the prognostic impact of time-to-complete adjuvant chemotherapy. Completing chemotherapy on time in the exactly prescribed schedule lead to the most favorable outcomes: complete response was more frequent in group completing at 105 days and compared favorably with patients having shortened intervals or delayed treatment administration.

Delays greater than 4 weeks significantly shortened PFS and OS, which may reflect overall health or clinical trajectory, for example, delays due to bowel obstruction or other hospitalization. Delays were not significantly associated with delay to initiation of chemotherapy. Shorter than expected adjuvant administration was associated with decreased survival, likely reflecting inability to complete all six cycles due to progressive disease or debility/illness, or progressive disease/platinum refractory determination. It is likely that the short interval groups reflect early determination of platinum-resistance and switches to second-line treatment.

An increasing number of weeks of delay progressively decreased the PFS/OS, arguing there is a relationship between sustained chemotherapy intensity and clinical outcome. The reported effect may be underestimated: long-term use of regimens containing bevacizumab or other maintenance therapies comprised 9.5% of the cohort. Because patients enrolled in clinical trials are selected for better performance status and tend to perform well on these regimens, these patients should have attenuated the apparent delay effect. Additional stratification by number of cycles received showed that patients receiving exactly 6 cycles have a progressive significant decrease in survival with progressive delays in chemotherapy administration, supporting the effect of chemotherapy delays affecting survival independent of the number of cycles received. While not significant due to small numbers, there is a strong potential effect of delay on survival after relapse. This effect was similar in magnitude for the patients stopping treatment early due to platinum resistance, with patients having on-time completion surviving 31.0 months after recurrence versus 19.3 months for patients with completion >105 days (p = 0.13). Further study will be required to determine whether delay patients have effects lasting in their subsequent intervals.

Strengths of our study include the large number of patients and the reliable data abstraction directly from infusion records. A hidden contribution is that we have demonstrated that an informatics team can directly assess and process this data, which may significantly reduce what would have been time- and labor-intensive manual abstraction. The present analysis of categorical and continuous treatment delay strengthens the body of evidence supporting a dose-response effect between delay and survival. Weaknesses of the current study include the retrospective nature, a limited number of non-serous histologies for subtype analysis, and the lack of control for unmodifiable risk factors such as age, BMI, and comorbidities. Furthermore, the cohort [12] has a limited number of patients who received neoadjuvant chemotherapy, despite the growing acceptance of this treatment strategy [13–15] hence these patients were excluded. Data is scant on the role of time off treatment for interval cytoreduction as a predictor of survival.

Overall, this study highlights the importance of keeping on-schedule for chemotherapy administration. Some of these delays are due to unmodifiable factors (bone marrow suppression and overall health status), but the survival effects highlight the importance of trying to keep the delays to a minimum and consider options of dose reduction and/or growth factor support as indicated instead of schedule delays. Models such as this can continue to be refined with neoadjuvant chemotherapy patients and provide personalized counseling to patients as they undergo treatment and begin post-treatment surveillance.

## Supporting information

S1 File Study patient characteristics. (XLS)
